# Bioactive Composite Methacrylated Gellan Gum for 3D-Printed Bone Tissue-Engineered Scaffolds

**DOI:** 10.3390/nano13040772

**Published:** 2023-02-19

**Authors:** Ugo D’Amora, Alfredo Ronca, Stefania Scialla, Alessandra Soriente, Paola Manini, Jun Wei Phua, Christoph Ottenheim, Alessandro Pezzella, Giovanna Calabrese, Maria Grazia Raucci, Luigi Ambrosio

**Affiliations:** 1 Institute of Polymers, Composites and Biomaterials, National Research Council, 80125 Naples, Italy; 2 Department of Chemical Sciences, University of Naples Federico II, 80126 Naples, Italy; 3 Bioelectronics Task Force, University of Naples Federico II, 80126 Naples, Italy; 4 Insectta, 60 Jalan Penjara, Singapore 149375, Singapore; 5 Department of Physics “E. Pancini”, University of Naples Federico II, 80126 Naples, Italy; 6 Department of Chemical, Biological, Pharmaceutical and Environmental Sciences, University of Messina, 98168 Messina, Italy

**Keywords:** gellan gum, hydroxyapatite, eumelanin, 3D printing, scaffolds, bone tissue engineering

## Abstract

Gellan gum (GG) was chemically modified with methacrylic moieties to produce a photocrosslinkable biomaterial ink, hereinafter called methacrylated GG (GGMA), with improved physico-chemical properties, mechanical behavior and stability under physiological conditions. Afterwards, GGMA was functionalized by incorporating two different bioactive compounds, a naturally derived eumelanin extracted from the black soldier fly (BSF-Eumel), or hydroxyapatite nanoparticles (HAp), synthesized by the sol–gel method. Different ink formulations based on GGMA (2 and 4% (*w*/*v*)), BSF-Eumel, at a selected concentration (0.3125 mg/mL), or HAp (10 and 30% w_HAp_/w_GGMA_) were developed and processed by three-dimensional (3D) printing. All the functionalized GGMA-based ink formulations allowed obtaining 3D-printed GGMA-based scaffolds with a well-organized structure. For both bioactive signals, the scaffolds with the highest GGMA concentration (4% (*w*/*v*)) and the highest percentage of infill (45%) showed the best performances in terms of morphological and mechanical properties. Indeed, these scaffolds showed a good structural integrity over 28 days. Given the presence of negatively charged groups along the eumelanin backbone, scaffolds consisting of GGMA/BSF-Eumel demonstrated a higher stability. From a mechanical point of view, GGMA/BSF-Eumel scaffolds exhibited values of storage modulus similar to those of GGMA ones, while the inclusion of HAp at 30% (w_HAp_/w_GGMA_) led to a storage modulus of 32.5 kPa, 3.5-fold greater than neat GGMA. In vitro studies proved the capability of the bioactivated 3D-printed scaffolds to support 7F2 osteoblast cell growth and differentiation. BSF-Eumel and HAp triggered a different time-dependent physiological response in the osteoblasts. Specifically, while the ink with BSF-Eumel acted as a stimulus towards cell proliferation, reaching the highest value at 14 days, a higher expression of alkaline phosphatase activity was detected for scaffolds consisting of GGMA and HAp. The overall findings demonstrated the possible use of these biomaterial inks for 3D-printed bone tissue-engineered scaffolds.

## 1. Introduction

In the last decades, hydrogels have proven to be an important class of biomaterial-based inks for bone tissue engineering applications, providing better flexibility, achieving an improved accuracy, and allowing the fabrication of scaffolds with defined shapes and with controlled and interconnected tailored porosity from a computer-aided design (CAD) file [1,2,3]. In particular, hydrogels based on naturally derived polymers, such as polysaccharides, are widely used due to their high-water content and the ability to structurally mimic the natural extracellular matrix (ECM) [1,4]. Despite their biocompatibility and biodegradability, poor mechanical properties along with a lack of inherent self-supporting abilities limit their use in three-dimensional (3D) printing applications. Therefore, the natural polymers’ printing fidelity in layer-by-layer approaches is not fully ensured; hence the need to be functionalized. Indeed, the introduction of suitable functional groups (i.e., by esterification, aminated carboxymethylation or methacrylation) may improve the mechanical stability after printing as well as the physico-chemical properties (i.e., residence time and swelling) of the final structure [5,6,7,8,9].

Among the polysaccharides, gellan gum (GG) has shown a broad perspective of applications due to its interesting inherent physico-chemical properties, excellent stability and biocompatibility [10]. GG is a linear negatively charged exopolysaccharide, produced by bacteria of the *Sphingomonas* strain. It consists of a linear tetrasaccharide repeating unit, including 1,3-β-D-glucose, 1,4-β-D-glucose, 1,3-β-D-glucuronic acid, and 1,4-α-L-rhamnose. Two acyl substituents O-acetate and L-glycerate, separately linked at the C-6 and C-3 positions of the same glucose unit, are present in GG’s natural form. GG dissolves in hot water assuming a “random coil” state and, when the temperature decreases, it self-organizes into a “double helix” structure, which is further stabilized by the presence of divalent cations [11,12]. GG and its derivatives have usually been employed as viscous enhancers [13] and in combination with other synthetic or natural polymers, such as starch [14], chitosan [15], methacrylated gelatin [16] and alginate [17], for regenerative medicine. As a biomaterial ink, it has many advantages over other hydrogels, including a high gelling efficiency at physiological temperature and a reasonable production cost [18]. However, physically crosslinked GG hydrogels lose their stability under physiological conditions, as, in this case, the divalent cations are replaced by monovalent ones that are present at much higher concentrations in vivo [12]. The stability and mechanical properties of GG can be enhanced by covalently conjugating carboxylic acid or hydroxyl groups with chemical crosslinkers [19,20,21]. In particular, the use of photo-polymerization as an alternative method for GG hydrogel formation, with increased structural and mechanical integrity, has triggered a considerable interest in the last decade. Indeed, for biofabrication purposes, the GG chain is commonly functionalized with methacrylate moieties to allow photocrosslinking, in the presence of a photoinitiator, upon exposure to ultraviolet (UV) light, enabling mechanical tunability of the hydrogel. Glycidyl methacrylate and methacrylic anhydride have been widely employed to produce methacrylated GG (GGMA) [13,21,22,23,24,25,26,27]. Recently, Jongprasitkul et al. [22] proposed a printable GGMA-based ink employing a combined ionotropic and photoinduced crosslinking. GGMA (2% (*w*/*v*)) with calcium chloride (90 mM) provided a formulation with the best printability. Indeed, the authors obtained consistent fibers even though the 3D-printed structures still lacked high resolution. However, apart from the geometrical constraints, a series of biological signals are essential to guide bone tissue regeneration at a cellular level. The most popular approach lies in the incorporation of inorganic micro-/nanoparticles, such as hydroxyapatite (HAp), within the hydrogel matrix, simultaneously acting as mechanical reinforcement and bioactive signals, promoting cell adhesion, proliferation and osteogenic differentiation in vitro and/or in vivo [28,29]. Moreover, ions released from HAp-embedded GG-based materials, particularly calcium ions, could also potentially be exploited to promote the hydrogels’ gelation [8]. Traditionally, nanocomposite scaffolds have been obtained with different strategies involving the physical mixing of HAp in natural or synthetic polymer matrices. Among the different technologies to produce HAp, the sol–gel method has emerged as a valid approach, as it allows a highly pure HAp to be obtained at low temperature and without using toxic solvents [25]. Over the past few years, considerable interest has been focused on the design of innovative hydrogels incorporating biomaterials and/or nature-inspired materials with the aim of improving the interaction of the scaffold with the bone tissue. In this regard, recently, particular attention has been devoted to the role played by eumelanins as bioactive agents. Eumelanins are the dark insoluble pigments found in mammalian skin, eyes and hair [30,31,32]. Thanks to a unique set of physico-chemical properties, eumelanins have found many applications as a functional biomaterial in different sectors, from organic (bio)electronics [33,34,35,36] to energy storage [37], photo- and radio-protection and regenerative medicine [38,39]. Novel insights indicate that eumelanin can specifically support bone tissue engineering [40,41]. Specifically, eumelanin has been highlighted to enhance the physico-chemical features of bone substitutes by stimulating stem cell and osteoblast functions and promoting the biomineralization process [40]. Yoo et al. were some of the first to study the time-dependent effects of melanin extracted from *Gallus gallus domesticus* on bone formation via osteoblast differentiation and the inhibition of osteoclast activity [40]. In particular, the authors provided in vitro evidence that the extract of melanin promoted osteoblast differentiation by activating bone morphogenetic protein 2 (BMP-2), small mothers against decapentaplegic homologs 5 (SMAD5), runt-related transcription factor 2 (RUNX2) signaling and regulating the transcription of osteogenic genes [40]. In a recent paper, D’Amora et al. showed the great potential of the eumelanin extracted from the black soldier fly (BSF-Eumel) in the design of 3D-printed bone tissue-engineered scaffolds based on methacrylated hyaluronic acid. These scaffolds showed the ability to promote the osteogenic differentiation of human mesenchymal stem cells (hMSCs) [41]. Prompted by these positive results, the study was extended to another type of 3D-printed scaffold based on GGMA. 

Herein, the main driving idea of this study was the design and characterization of bioactivated GGMA-based scaffolds by the 3D printing technique. To this aim, GGMA was synthesized by the covalent linking of methacrylic moieties to GG and further functionalized by physical blending with HAp, produced via the sol–gel route, or BSF-Eumel, to obtain the GGMA-based inks. Bearing this in mind, the novelty of the work relies on the simultaneous combination of natural-based biomaterials and optimized-processing conditions to provide a higher-value-added product, from nature to regenerative medicine. The influence of BSF-Eumel and HAp on GGMA processability as a biomaterial ink was analyzed. Furthermore, the physico-chemical properties of the 3D-printed scaffolds together with their morphological and mechanical performances were evaluated. Meanwhile, the biological behavior of the 3D-printed scaffolds, in terms of proliferation and bone differentiation was assessed in vitro on osteoblast cells.

## 2. Materials and Methods

### 2.1. Chemicals

Low acyl GG (Gelzan), methacrylic anhydride (MA, purity ≥ 94%), sodium hydroxide (NaOH, reagent grade, 98%), 2-hydroxy-4′-(2-hydroxyethoxy)-2-methylpropiophenone (Irgacure 2959, gas chromatography area ≥ 98%), and absolute ethanol (EtOH, purity ≥ 99.9%) were obtained from Sigma Aldrich (Milan, Italy) and were used for GGMA synthesis.

Calcium nitrate tetrahydrate (Ca(NO_3_)_2_4H_2_O, ACS reagent, 99%), diammonium hydrogen phosphate ((NH_4_)_2_HPO_4_, ACS reagent Ph Eur.), ammonium hydroxide (NH_4_OH, ACS reagent, 28.0–30.0% NH_3_ basis), phosphate-buffered saline (PBS), were from Sigma Aldrich (Milan, Italy) and were used for HAp synthesis.

Lactic acid (C_3_H_6_O_3_), hydrochloric acid (HCl, ACS reagent, 37%), were from Sigma Aldrich (Singapore) and were used for BSF-Eumel extraction.

Calcium chloride (CaCl_2_, ≥ 96.0%) was from Sigma Aldrich (Milan, Italy) and was employed for 3D-hydrogel preparation. Fresh deionized (*di*H_2_O) water (for chromatography LC-MS Grade, conductance at 25 °C ≤ 1 µS/cm, Sigma Aldrich, Milan, Italy) was used throughout the chemical synthesis, while distilled water (*d*H_2_O) was employed for purification steps and characterization studies.

Murine osteoblast-like cell line (7F2, CRL-12557) was obtained from the American Type Culture Collection (ATCC, Manassas, VA, USA) and adopted for scaffold in vitro validation studies. Dulbecco’s Modified Eagle Medium high glucose with and without Phenol Red (DMEM-HG) were from Gibco by Thermo Fisher Scientific (Monza, Italy). Dimethyl sulfoxide (DMSO, purity ≥ 99.9%), formalin solution (neutral buffered 10%), fetal bovine serum (FBS), antibiotic/antimycotic solution (100×), L-glutamine (200 mM), trypsin-EDTA solution (Tryp-EDTA, 1×) and 4′,6-diaminidin-2-fenilindolo (DAPI) were all from Sigma Aldrich (Milan, Italy) and were used for cell maintenance and in vitro studies. Red Cell Tracker Red CMTPX was from Thermo Fisher Scientific (Milan, Italy) and was used for fluorescence microscopy studies.

### 2.2. Synthesis and Characterization of Photocrosslinkable GGMA

**Figure 1:**
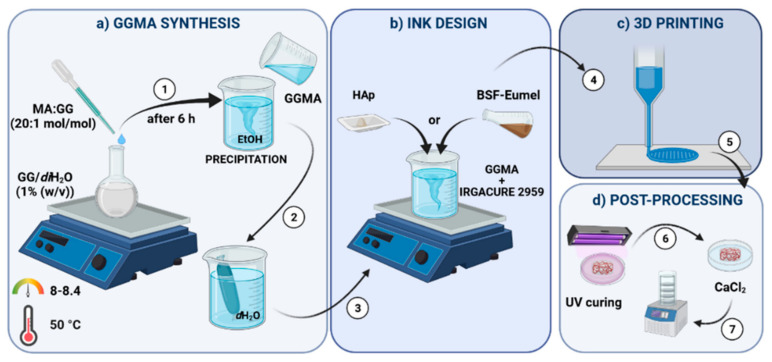
Schematic representation of the multi-step methodology adopted for the bioactivated methacrylated gellan gum (GGMA)-based scaffold fabrication by 3D printing. The methodology includes: (**a**) GGMA synthesis, (**b**) ink design, (**c**) 3D printing and (**d**) post-processing.

GGMA was synthesized via substitution of the hydroxyl groups in the GG repeating units with MA. Briefly, low acyl GG was dissolved in *di*H_2_O at 1% (*w*/*v*) in a round-bottom flask and heated at 90 °C overnight under constant stirring, to achieve a complete dissolution. The material was functionalized by reacting with MA at 50 °C, by adapting Coutinho et al.’s protocol [26], as reported in Figure 1. A 20 mol % MA excess per unit of GG was used for the methacrylation reaction. The pH of the solution was kept between 8 and 8.4 by adding 5M NaOH, to balance the presence of methacrylic acid as byproduct of the reaction, preventing the gelification of GG in acidic conditions. After 6 h reaction, GGMA was drop-wise precipitated in cold absolute EtOH under vigorous magnetic stirring. An EtOH:GGMA solution volume ratio of 4:1 was used. The final solution was filtered, the collected modified polymer was re-solubilized in *di*H_2_O and subsequently purified by dialysis against *d*H_2_O for 72 h, using dialysis membrane tubes (12–14 kDa molecular weight cut-off; Sigma Aldrich, Milan, Italy) to ensure complete removal of MA and EtOH residues. During the dialysis, *d*H_2_O was refreshed at least twice a day. The material was lyophilized (LaboGene’s CoolSafe 55-4 PRO, Bjarkesvej, Denmark) and stored at −80 °C for further use.

Attenuated total reflection Fourier transform infrared (ATR-FTIR) spectroscopy (Thermo Fisher Nicolet IS10, Waltham, MA, USA) was employed to assess the GG functionalization postmethacrylation reaction. The chemically modified polymer was scanned from 500 to 4500 cm^−1^ with a resolution of 2 cm^−1^. The neat GG under the same conditions was used as control.

### 2.3. Preparation and Characterization of GGMA-Based Biomaterial Inks

In the present study, the term ink (in short, “ink”) refers to the different formulations without cells.

*GGMA ink*. GGMA was dissolved in *di*H_2_O at 70 °C for 2 h, at two different concentrations 2 and 4% (*w*/*v*). Afterwards, Irgacure at 5% concentration (w_Irgacure_/w_GGMA_) was added in the dark and magnetically mixed until a homogeneous ink formulation was achieved.

*GGMA/BSF-Eumel ink*. GGMA/BSF-Eumel ink was obtained by physical blending of GGMA and BSF-Eumel. Eumelanin extracted from black soldier fly (BSF) cuticles (Insectta Pte. Ltd. UEN: 201809941M International Publication No.: WO/2021/183058 A1) was kindly provided by Insectta, as a sub-micrometer colloidal particle suspension. Black soldier fly (*Hermetia illucens*) pupal exuviae was obtained from Hermetia Bio Science (Jakarta, Indonesia) and ground into approximately 0.5 mm pieces using a blender (Robot Coupe Blixer 4, Vincennes, France). Mineral removal was performed by using 10% (*w*/*w*) C_3_H_6_O_3_, whilst protein removal was performed by using 1 M NaOH. A 3M NaOH solution was used to dissolve melanin. The melanin-containing supernatant was then filtered, and melanin was precipitated with 37% (*w*/*v*) HCl. The melanin was separated by centrifugation, washed, and subjected to lyophilization to produce salt-free and water-soluble particles. For GGMA/BSF-Eumel ink production, a preliminary study allowed the selection of the optimal BSF-Eumel working concentration [39]. To this aim, GGMA (2 and 4% (*w*/*v*)) was dissolved in *di*H_2_O in a lower volume, containing Irgacure, and the complementary volume of BSF-Eumel, diluted in *di*H_2_O, was directly added to the polymer solution, to reach the final concentration of 0.3125 mg/mL. The solution was mixed at room temperature, until a uniform yellowish hydrogel was formed [41].

*GGMA/HAp nanocomposite ink*. GGMA/HAp nanocomposite ink was prepared by physical blending of GGMA and HAp. HAp was synthesized at room temperature by a sol–gel method [42], starting from Ca(NO_3_)_2_·4H_2_O and (NH_4_)_2_HPO_4_. The procedure consisted of adding drop-wise a 3.58 M water solution (1.67 mL) of (NH_4_)_2_HPO_4_ (P-solution) to a 3 M water solution (3.33 mL) of Ca(NO_3_)_2_·4H_2_O (Ca-solution) in order to obtain a Ca/P mol ratio of 1.67; then, the pH of the mixture was adjusted up to pH 9 by adding NH_4_OH. The HAp solution was gently shaken at 100 rpm and 37 °C until gelification occurred. The obtained gel phase was dialyzed in 0.01 M PBS at pH 7.4 to achieve a stable physiological pH and subsequently lyophilized. HAp was completely characterized from a morphological, physico-chemical and biological point of view as reported in Raucci et al. [42]. For GGMA/HAp nanocomposite ink preparation, GGMA (2 and 4% (*w*/*v*)) was dissolved in *di*H_2_O, containing Irgacure, 10 and 30% (w_HAp_/w_GGMA_) HAp was added. The formulations were vigorously stirred for 30 min to obtain a homogeneous particle distribution.

Starting from now and throughout the manuscript, the different GGMA-based ink formulations will be identified as: GGMAX, GGMAX/BSF-Eumel and GGMAX/HApY, where X stands for the (% (*w*/*v*)) of GGMA and Y represent the (% (w_HAp_/w_GGMA_)) of HAp, respectively. However, GGMA, GGMA/BSF-Eumel as well as GGMA/HAp will be used along the manuscript referring to the biomaterial ink, in general.

*Qualitative printability assessment*. The printability of GGMA2- and GGMA4-based inks was assessed by filament fusion test. The neat and composite inks were loaded in a Luer-lock glass syringe (5 mL) with 15.5 mm inner diameter, by using a “Rokit Invivo 4D2” (Rokit Healthcare Inc., Seoul, Republic of Korea) and a 1.80 firmware. Four different line patterns were considered, each one characterized by a specific infill of 25, 35, 45 and 55%. The printing speed was set at 3 mm/s. The dispenser temperature was set at 10 and 35 °C, for GGMA2- and GGMA4-based inks, respectively. Pictures were obtained using a Nikon digital camera directly after printing.

### 2.4. Preparation and Characterization of GGMA-Based Scaffolds

The 3D printing was performed through “Rokit Invivo 4D2”. The input printing model was sliced using NewCreatorK 1.57.70 (Rokit Healthcare Inc., Seoul, Republic of Korea). Printing speed and temperature were set as for the fusion test. A needle with 0.41 mm diameter (Gauge 27), 0.3 mm layer thickness, 35 and 45% of infill density, according to the filament fusion test results, was used to build cylindrical samples with 13 mm diameter and 3 mm thickness. Horizontal and vertical struts were alternated with each layer following a grid pattern. After printing, scaffolds were chemically crosslinked by exposure to UV light (Analytik Jena UVP crosslinker, Jena, Germany, λ: 365 nm, P: 10 J/cm^2^) for 10 min. After that, a further physical crosslinking step was performed by dipping the scaffolds for 10 min, in 0.05% (*w*/*v*) CaCl_2_ at room temperature [26,34,43]. A schematic summary of the process is depicted in Figure 1, including the polymer modification, the ink formulation design, the 3D printing and the post-processing.

*Dynamic mechanical analysis*. TA-Q800 (TA-Instrument, New Castle, DE, USA) was employed to perform dynamic mechanical analysis (DMA) of the scaffolds. The frequency was set at 1.0 Hz, which simulates the physiological stride. Furthermore, an amplitude of 100 µm in compression, a preload of 0.001 N and a force track of 125% were adopted. The tests were carried out in a closed chamber in wet state, and at room temperature. Sample dehydration was negligible as the measurement procedure required only a few minutes. 

*Thermogravimetric analysis*. Thermogravimetric analysis (TGA) was performed to evaluate the thermal stability and the effective weight percentage of the components in the scaffolds using a TA Instruments TGA model 2950 (New Castle, DE, USA). Dried specimens (4–7 mg) were heated, under nitrogen (N_2_) flow, from 25 to 800 °C at a heating rate of 10 °C/min.

*Morphological analysis*. 3D-printed scaffolds were observed by scanning electron microscopy (SEM, FEI Quanta 200 FEG, Hillsboro, OR, USA). For sample preparation, scaffolds were washed with *d*H_2_O, frozen at −80 °C and lyophilized in a vacuum freeze dryer for 48 h. Subsequently, the lyophilized samples were coated with an ultrathin layer of Au/Pt by using an ion sputter and observed by SEM. In addition, a Nikon digital camera was used for optical imaging of the 3D-printed scaffolds.

*Swelling and stability studies*. Dried scaffolds were weighed (w_0_) and left to swell in sterile phenol red-free DMEM-HG supplied with antibiotics (pH 7.4, T = 37 °C, V = 5 mL, up to 28 days) mimicking the physiological conditions. To assess their retention capacity, the swollen hydrogels were then taken out at fixed time points, quickly blotted on a filter paper to remove the superficial adsorbed solution, the weight was recorded (w_t_) and the samples placed in medium again. The swelling ratio (Q) was calculated according to Equation (1):

(1) $$Q=\left(\frac{w_{t}-w_{0}}{w_{o}}\right)$$

For the stability tests, at fixed time points, samples were taken out, frozen at −80 °C, lyophilized and weighed (w_t_). The relative weight (W_r_) was obtained according to Equation (2):

(2) $$W_{r}=\frac{w_{t}}{w_{o}}$$

*Cell culture*. For the in vitro studies, murine osteoblast cells (7F2) were cultured in DMEM-HG supplemented with 10% (*v*/*v*) FBS, 1× antibiotic/antimycotic solution, and 2 mM L-glutamine, in a humidified atmosphere containing 5% CO_2_ and 95% air at 37 °C until about 70–80% confluence was achieved.

*Cell Viability*. For the assessment of 7F2 cell viability, a standard MTT (3-(4,5-dimethylthiazol-2-yl)-2,5-diphenyltetrazolium bromide) (Sigma Aldrich, Milan, Italy) assay was performed to measure cell metabolic activity. Prior to cell seeding, GGMA-based scaffolds were sterilized under UV light for 2 h (1 h per side), and 7F2 cells (8 × 10^5^ cells/scaffold) were seeded on the top of dried GGMA-based scaffolds in 50 μL medium volume. After 4 h, early cell adhesion was firstly evaluated, while additional fresh medium was drop-wise added to the resultant 7F2-seeded scaffolds (1 mL/well) for assessing the proliferative response at different time points (7-, 14- and 21-days post-seeding). At each time point, MTT dissolved in PBS was added to each well at the final concentration of 50 μg/mL and incubated at 37 °C for 3 h. The medium was removed and the purple formazan crystals, produced in viable cells by MTT reaction, were solubilized with 10% (*v*/*v*) DMSO (500 µL). Finally, the arbitrary units of absorbance of the obtained solutions (proportional to the metabolic activity of cells) were spectrophotometrically measured (λ = 570 nm) by UV–Vis spectrophotometer (Victor X3 Multilabel Plate Reader, Perkin Elmer, Milan, Italy). Absorbance values were normalized to the adhered cells after 4 h and expressed as percentage of cell viability.

Morphology of 7F2 cells seeded on GGMA4, GGMA4/BSF-Eumel and GGMA4/HAp30 was analyzed in a fluorescence microscope (JuLI Stage Real-Time CHR, Cell History Recorder, NanoEn TecK. Inc. (HQ), Seoul, Republic of Korea) 24 h after cell seeding. To this aim, cells were stained with a 50 μg red cell tracker and seeded onto the scaffolds. At 24 h post-seeding, cells were fixed with 10% (*v*/*v*) formalin solution and 10 μg/mL DAPI solution was added for 15 min at 37 °C to cells. The cells were washed three times with 1× PBS and observed by fluorescent microscope.

*Cell osteogenic differentiation by alkaline phosphatase expression*. The differentiation capability of 7F2 osteoblasts seeded on GGMA-based scaffolds was evaluated by measuring alkaline phosphatase (ALP; SensoLyte pNPP ALP assay kit, ANASPEC, Milan, Italy) activity at different time points (7, 14 and 21 days). The evaluation of ALP expression was performed on cell lysates (50 μL) obtained by treating the cells with 1× lysis buffer including 0.2% (*v*/*v*) of Triton X-100. The optical absorbance of cell lysates was recorded with a UV–Vis spectrophotometer at 405 nm. To correct the ALP values for the number of cells present on each scaffold, double stranded DNA (dsDNA) was measured using a PicoGreen_dsDNA quantification kit (QuantiFluor dsDNA System, Promega, Milan, Italy) following the manufacturer’s protocol. The fluorescence of PicoGreen was determined at 520 nm after excitation at 585 nm using a UV–Vis spectrophotometer. The results of ALP activity were reported as nanograms (ng) of ALP normalized to the micrograms (µg) of total DNA content.

### 2.5. Statistics and Data Analysis

For each experiment, at least triplicate specimens were tested, unless differently stated. Results are presented as mean ± standard error mean (S.E.M.) of independent measurements. Statistical analysis of variance of the means was assessed by one-way or two-way ANOVA followed by Tukey’s post hoc test with multiple comparisons between the different groups, using GraphPad Prism software (version 7.0). Different levels of significance were considered 95–99.9999% (* *p* < 0.05, ° *p* < 0.01, ^#^ *p* < 0.001, ^§^ *p* < 0.0001) among the different results.

## 3. Results

3D-printed scaffolds consisting of bioactive GGMA-based ink formulations, embedding BSF-Eumel or HAp, were successfully realized, showing tunable physico-chemical, mechano-structural and biomimetic properties. Over the past years, GG and GGMA have been widely employed to fabricate scaffolds intended for regenerative medicine with conventional fabrication techniques [29,44,45]; whereas, recently they have gained an increased interest for 3D printing applications [22,46].

### 3.1. GG modification and Characterization

**Figure 2:**
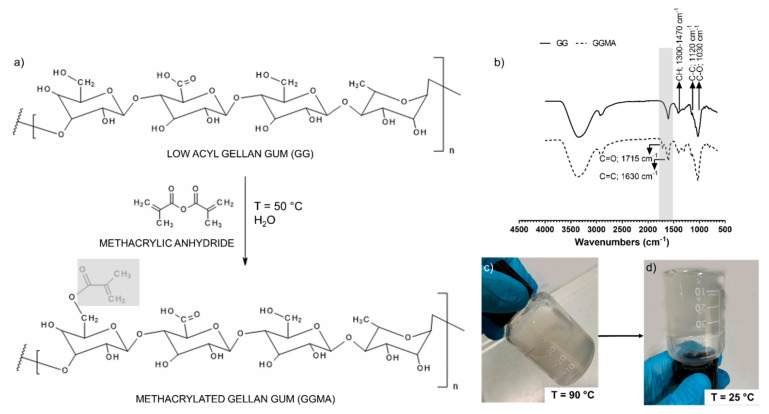
(**a**) Schematic illustration of the methacrylation reaction between low acyl gellan gum (GG) and methacrylic anhydride (MA) to synthesize methacrylated gellan gum (GGMA). (**b**) ATR-FTIR spectra of GG (top) and GGMA (bottom). (**c**) Representative photograph of GGMA solution at 90 °C and (**d**) at 25 °C, showing the system’s turbidity and the transition phase from liquid to gel state.

A simple scheme of the GG methacrylation chemical synthesis is illustrated in Figure 2a. The successful modification of GG was assessed by ATR-FTIR. Figure 2b reports the ATR-FTIR spectra of unmodified GG (Figure 2b top) and GGMA (Figure 2b bottom). Both spectra display a broad −OH stretching band above 3000 cm^−1^, and skeletal vibration involving the C–O stretching band at 1030 cm^−1^, which are typical for all polysaccharides. The bands at 1120 and 1300–1470 cm^−1^ are due to C–C stretching and C–H bending, respectively. The characteristic double bond band observed in GGMA spectra at 1630 cm^−1^ is due to the C=C stretching typical of the methacrylic moieties in the GG, while the absorption band at 1715 cm^−1^, ascribable to the carbonyl (C=O) stretching, confirms the chemical modification of GG. Due to the moderately hydrophobic nature of the methacrylic moieties, the synthesized GGMA was not fully soluble in water, forming a slightly turbid solution, as shown in Figure 2c,d [47]. Furthermore, from Figure 2c,d, the sol–gel transition is evident, by decreasing the temperature from 90 to 25 °C.

### 3.2. Qualitative Printability Assessment of GGMA-Based Ink

**Figure 3:**
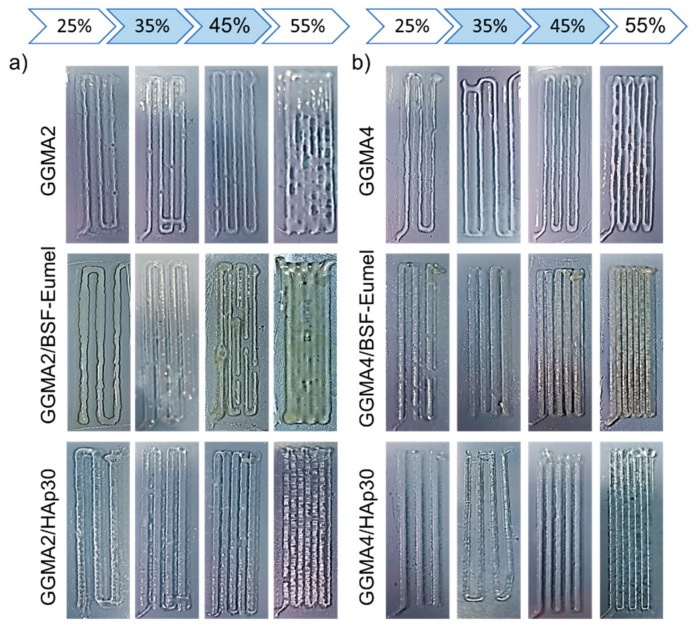
Filament fusion test performed on (**a**) GGMA2-based and (**b**) GGMA4-based ink formulations, by varying the percentage infill from 25 to 45%.

The printability of ink formulations critically depends on their rheological properties, and, hence, their mechanical behavior [5]. With this aim, GGMA-based ink formulations (GGMA2 and GGMA4-based inks) were screened with a qualitative fusion test to ensure their printability (Figure 3). Results from the filament fusion test showed that GGMA4-based ink formulations were characterized by a better consistency (Figure 3b) when compared to GGMA2-based ones (Figure 3a). Indeed, GGMA4 inks allowed a continuous strut deposition to be obtained, ensuring good printability (Figure 3b). By further increasing the concentration of GGMA, the printability could be seriously compromised, producing brittle and non-printable inks. The presence of BSF-Eumel and HAp improved the printability of the ink, especially at the highest GGMA concentration, as highlighted by a more defined fiber. In terms of infill, results showed that for all the formulations, there was a distinct relationship between the evolution of filament-gap closure and the infill increase. Indeed, filaments deposited with a 55% infill showed a clear overlap. For this reason, 35 and 45% infills were selected as the best parameters for performing further studies (Figure 3) [5].

### 3.3. Three-Dimensional printing and Characterization of GGMA-Based Scaffolds

Both GGMA-based concentrations were printable and allowed obtaining 3D-printed scaffolds, by using selected percentage infills (35 and 45%). The printed scaffolds were then post-processed, with a two-step crosslinking involving UV and CaCl_2_, to enable geometric shape retention over time. The presence of cations in the solution and their effect on the hydrogel network’s entanglement strongly influenced the scaffold’s response, in terms of swelling behavior and mechanical properties, as also assessed by Coutinho et al. [26].

**Figure 4:**
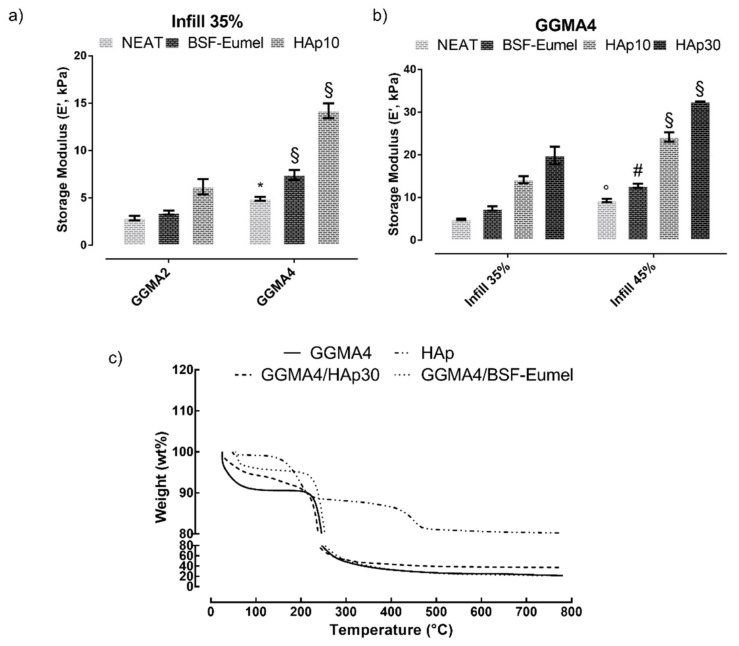
(**a**) GGMA concentration (2–4% (*w*/*v*)) effect on the storage modulus, by dynamic mechanical analysis (DMA), fixing the infill at 35% among the different scaffolds. (**b**) Infill effect on the storage modulus of GGMA-based scaffolds, by DMA, fixing the GGMA concentration at 4% (*w*/*v*). (**c**) Thermogravimetric analysis of HAp and GGMA4-based scaffolds performed under N_2_ flow, heating from 25 to 800 °C at 10 °C/min. Statistical analysis of variance on mean values was assessed by one-way ANOVA followed by Tukey’s post hoc test with multiple comparisons. (Histograms (mean value ± S.E.M.) with ^*^ *p* < 0.05, ^°^ *p* < 0.01, ^#^ *p* < 0.001, ^§^ *p* < 0.0001).

*Dynamic mechanical analysis*. DMA aimed to assess the effect of the polymer concentration (fixing the infill at 35%, Figure 4a) and the influence of the infill percentage (fixing the polymer concentration, Figure 4b) on the storage modulus (E’) of GGMA-based scaffolds. In addition, the effect of BSF-Eumel and HAp on the storage modulus at different concentrations was also assessed. Mechanical properties were proven to be strongly influenced by the polymer concentration (Figure 4a). In general, GGMA4-based scaffolds showed E’ values higher than GGMA2. BSF-Eumel did not significantly improve the mechanical performances of the GGMA scaffolds. Indeed, E’ ranged between 2.8 and 3.5 kPa, for GGMA2 and between 4.9 and 7.4 kPa for GGMA4. However, by increasing the HAp amount up to 10% (w_HAp_/w_GGMA_), E’ values increased from 2.9 to 6.2 kPa (for GGMA2, ^§^ *p* < 0.0001) and from 4.9 to 14.2 kPa (GGMA4, ^§^ *p* < 0.0001) (Figure 4a and b). Similarly, the geometrical constraints also affect the mechanical behavior. Therefore, the effect of the percentage infill on these scaffolds was analyzed (Figure 4b). It was observed that by increasing the infill up to 45%, E’ increased from 4.9 to 9.3 kPa (GGMA4), from 7.4 to 12.7 kPa (GGMA4/BSF-Eumel) and from 14.1 to 24.2 kPa (GGMA4/HAp10). Regarding GGMA4/HAp at 45% infill, by increasing HAp concentration from 10 to 30% (w_HAp_/w_GGMA_), E’ showed a significant enhancement from 24.2 to 32.5 kPa (^§^ *p* < 0.0001), respectively. In conclusion, scaffolds printed by using GGMA-based inks with the highest polymer concentration (4% (*w*/*v*)) and the highest percentage infill (45%) were selected for the following physico-chemical and biological characterization. This choice was based on the compromised results arising from the printability assessment (i.e., filament fusion test) and the mechanical behavior (i.e., DMA test). On one hand, printability with a GGMA concentration higher than 4% (*w*/*v*) might be seriously compromised. Furthermore, the fusion test highlighted that over 45% infill, the fibers overlapped, resulting in a 3D non-porous structure. On the other hand, DMA analysis allowed the selection of scaffolds with mechanical behavior suitable for the desired biomedical application [44].

*Thermogravimetric analysis*. The thermal properties of GGMA and bioactivated GGMA-based 3D-printed scaffolds were studied through non-isothermal TGA, by evaluating the variations of residual mass percentage with the temperature increases (Figure 4c). The mass loss curves for all the samples exhibited a similar pattern of thermal behavior. The GGMA4-based scaffold thermograms show two main weight loss stages: an initial mass reduction (~10 wt%) in the temperature range 50–100 °C, which is related to the dehydration of physically and chemically adsorbed water and a remarkable mass change (~60 wt% for GGMA4 and GGMA4/BSF-Eumel, ~48 wt% for GGMA4/HAp30) in the temperature range 200–400 °C, represented by a sharp curve regimen with least slope corresponding to the organic breakdown of polysaccharide GG chains [39]. The GGMA4 residual mass at 800 °C (~20 wt%), indicated by the presence of a weight-loss plateau, is caused by its incomplete combustion in the absence of oxygen [48]. The GGMA4/BSF-Eumel residual mass at 800 °C (~21 wt%) is not significantly different compared to GGMA4, whereas the GGMA4/HAp30 residual mass at 800 °C (~37 wt%) corroborates the theoretical amount of HAp (30% (w_HAp_/w_GGMA_) settled during the synthesis and reported in a previous study [49]. In addition, the overall TGA results confirmed that the degradation temperature is higher than the working temperature selected for the 3D printing process. Furthermore, the presence of HAp was not affected by the process, highlighting that the HAp did not settle down in the syringe during the 3D printing process and remained well dispersed.

**Figure 5:**
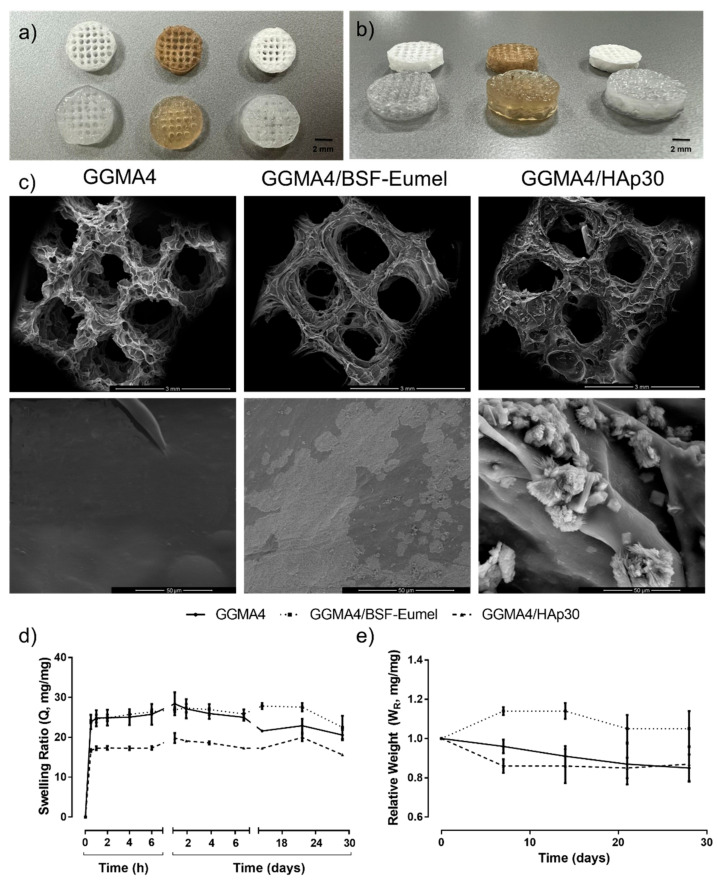
Optical images of dry (top row) and wet (bottom row) GGMA4 (left), GGMA4/BSF-Eumel (middle) and GGMA4/HAp30 (right) 3D-printed scaffolds with 45% infill (scale bar: 2 mm): (**a**) top view and (**b**) lateral view. (**c**) SEM micrographs of GGMA4 (left), GGMA4/BSF-Eumel (middle) and GGMA4/HAp30 (right) 3D-printed scaffolds with 45% infill (Mag. 50× and scale bar: 3 mm (top row); Mag. 2000× and scale bar: 50 μm (bottom row)). (**d**) Swelling ratio (Q, expressed as a dimensionless ratio) and (**e**) relative weight (W_R_, expressed as a dimensionless ratio) of GGMA4, GGMA4/BSF-Eumel and GGMA4/HAp30 3D-printed scaffolds as function of time in DMEM-HG supplemented with antibiotics up to 28 days in physiological conditions (pH 7.4 at 37 °C).

*Morphological analysis*. A porous microstructure is a key requirement for 3D scaffolds, which ensures the cell growth and assists the transport of essential nutrients. The porous structure of GGMA-based scaffolds was evaluated in terms of pore spatial distribution, size, shape and interconnection degree, as reported in Figure 5a,c, by qualitative observation via digital camera and SEM. A good shape retention with minimal shrinkage within the scaffold macrostructure was observed. This could be due to a loss in the liquid content following the crosslinking and freeze-drying processes. However, GGMA4-based scaffolds held their lattice-like structure with a uniform pore distribution, without collapsing in the z-direction (Figure 5a,b). The scaffolds presented a high and interconnected macroporosity, clearly reflecting the CAD design and confirming a good shape fidelity. Furthermore, SEM micrographs of GGMA4-based scaffolds at higher magnification revealed the presence of HAp on the surface of the scaffold, whereas BSF-Eumel appeared distributed in the GGMA matrix (Figure 5c). This evidence was also supported by ATR-FTIR spectra (Figure S1) showing the signals relative to HAp in the GGMA4/HAp scaffold to be more intense with respect to those of GGMA4, suggesting that HAp was present as an external coating. In contrast, in the case of the GGMA4/BSF-Eumel scaffold, the signals of GGMA4 and BSF-Eumel exhibited comparable intensities indicative of a homogeneous distribution of the melanin within the ink. This aspect is of paramount importance as it can differently and simultaneously influence cell adhesion and differentiation, due to variations in the surface area and the easy accessibility of the bioactive signals.

*Swelling and stability studies*. Scaffold swelling and stability properties provide an indirect measure of the GGMA4-based scaffold crosslinking density. These features might influence the scaffold microstructure and the mechanical stability, modulating the diffusion of nutrients and cell motility with the 3D matrix. The swelling ratio (Q) of GGMA4-based scaffolds, assessed in physiological conditions (phenol red-free DMEM-HG, pH 7.4 at 37 °C) and calculated according to Equation 1, is shown in Figure 5d. GGMA-based scaffolds reached the highest swelling ratio at 24 h (28.4 ± 2.9 for GGMA4, 26.9 ± 0.6 for GGMA4/BSF-Eumel and 19.8 ± 1.3 for GGMA4/HAp30, ^§^ *p* < 0.0001), holding the swollen state up to 28 days. It was reported that ions might induce the double helix formation and the establishment of junction zones, resulting in more crosslinked networks [23]. In addition, GGMA4 and GGMA4/BSF-Eumel scaffolds showed a similar swelling profile without a statistically significant difference. Conversely, HAp seemed to induce lower water absorption owing to reduced chain mobility. However, neat GGMA highlighted a significant tendency to de-swell after 7 days, while GGMA4/BSF-Eumel and GGMA4/HAp30 exhibited this behavior over longer times (21–28 days). The different swelling behaviors could be ascribed to the presence of HAp nanoparticles and BSF-Eumel sub-micrometer particles that stabilized the structures. For neat GGMA, due to the higher swelling ratio, it is possible that small fragments of GGMA were released during the swelling, and this may explain the de-swelling behavior and the reduction in weight registered for the GGMA samples in the first 10 days of soaking in DMEM-HG.

The hydrolytic degradation behavior, assessed through stability studies, according to Equation 2, aimed at recapitulating the in vitro physiological conditions (phenol red-free DMEM-HG at 37 °C up to 28 days without enzymatic components, Figure 5e). GGMA4 and GGMA4/HAp30 scaffolds lost almost 10% of their mass in 28 days. GGMA4 exhibited a progressive slow weight loss over time, whereas GGMA4/HAp30 showed an initial burst weight decrease, which remained constant up to 4 weeks. By contrast, GGMA4/BSF-Eumel scaffolds proved to be more stable. This may be ascribed to the presence of negatively charged groups, mainly carboxyl groups but also catechols, along the eumelanin backbone [31]. These groups provide an array of chemical sites for the attraction and chelation of metal cations from the medium, which in turn are partially available to stabilize the negatively charged polymer of the scaffold, in a process similar to ionic bond-based crosslinking [50].

**Figure 6:**
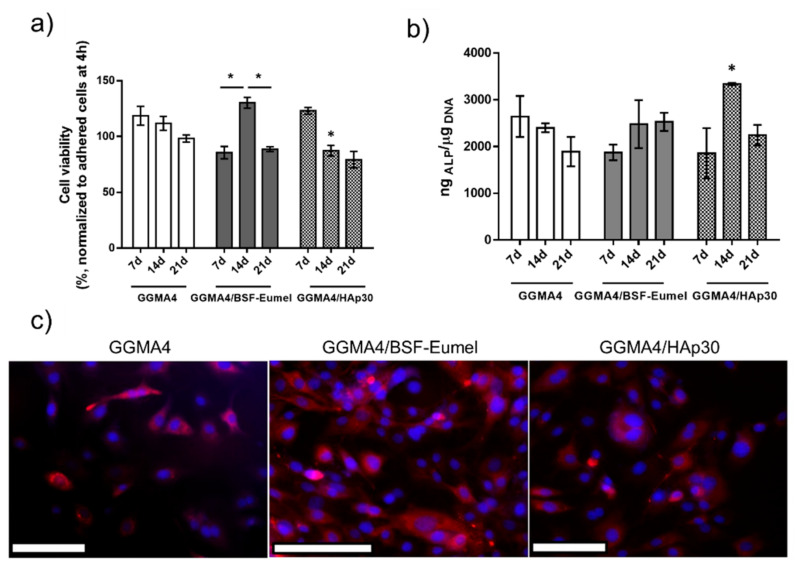
(**a**) Cell viability of 7F2 osteoblasts seeded onto GGMA4, GGMA4/BSF-Eumel and GGMA4/HAp30 scaffolds at 7, 14 and 21 days of cell culture normalized to adhered cells 4 h post-seeding (data are reported as mean value of cell viability percentage ± S.E.M., n = 4, * *p* < 0.05 vs. GGMA4). (**b**) ALP activity (expressed as ng_ALP_/μg_DNA_) for GGMA4, GGMA4/BSF-Eumel and GGMA4/HAp30 at 7, 14 and 21 days (data are reported as mean value ± S.E.M., n = 4, * *p* < 0.05 vs. GGMA4. (**c**) Representative fluorescent images acquired of 7F2 cells seeded on the different structures, at 24 h after cell seeding. (Scale bar: 500 μm).

*Biological analysis*. The inherent structural similarities to native glycosaminoglycans and, hence, the proven biocompatibility, along with the mild processing conditions for scaffold fabrication, have made GG-derivatives suitable for tissue engineering applications [51]. The properties of GG-derivatives, such as mechanical stability, flexibility and responsiveness to external stimuli, have been improved and tailored by introducing bioactive moieties including nanoparticles and bioactive compounds [6]. Therefore, this work aimed to study the effect of BSF-Eumel and HAp as bioactive signals in triggering a physiological response in 7F2 osteoblastic cells when seeded on a 3D-printed GGMA-based scaffold. Cell proliferation and early osteogenic differentiation at different time points of 7, 14 and 21 days were analyzed. HAp-functionalized GGMA composites have recently been investigated, proving their capability to promote human adipose-derived stem cell adhesion and spreading within the GGMA/HAp spongy-like hydrogels [44], whereas there are still few studies in the literature focused on the effect of BSF-Eumel in addressing a specific physiological response when grown on a GGMA-based matrix. Therefore, the GGMA/BSF-Eumel composites herein proposed may be held among the first attempts. As widely reported in the literature, the osteoinductive features of HAp in inducing hMSC differentiation towards osteoblast-like phenotype and supporting differentiated osteoblast cells towards bone matrix formation are well-known [39]. Likewise, eumelanin is raising a great interest in biomedical applications for its inherent physico-chemical properties, which can promote natural regenerative processes [38,52,53]. The viability of 7F2 osteoblast cells (expressed as cell viability percentage normalized to adhered cells at 4 h) and alkaline phosphatase activity (expressed as ng_ALP_/µg_DNA_), after 7, 14 and 21 days of culture, are shown in Figure 6a,b, respectively. The percentage of cell viability over 98% was measured for 7F2 osteoblast cells up to 21 days in GGMA4 scaffolds, showing that the neat scaffold was able to sustain 7F2 survival but not proliferation (Figure 6a), due to the lack of anchorage points in the gellan gum, which are essential for cell spreading and cellular infiltration [54]. To overcome this limitation, HAp and BSF-Eumel were used and investigated as bioactive signals to improve cell adhesion and proliferation. ANOVA showed that there was no significant difference in the cell viability of GGMA4 over time (7, 14 and 21 days, * *p* < 0.05). This was also qualitatively assessed by fluorescence microscopy imaging, revealing that osteoblasts adhered to the polymeric matrix, organising their cytoskeleton and exhibiting a typically embossed cell shape when dispersed within the 3D matrix (Figure 6c), as also seen by da Silva et al. [55].

Therefore, the study of BSF-Eumel and HAp effect as bioactive signals was extremely interesting for tailoring the GGMA features. GGMA/BSF-Eumel showed the highest cell proliferation compared to the other scaffolds (Figure 6a). Indeed, GGMA4/BSF-Eumel induced an increase in 7F2 osteoblast cell proliferation, achieving a maximum at 14 days (130.2 ± 12.6%, * *p* < 0.05) (Figure 6a). In contrast, 7F2 cells seeded onto GGMA4/HAp30 reached their maximum proliferation already at 7 days (123.0 ± 6.7%), with GGMA4/HAp30 acting as a booster for cell proliferation stimulus. A subsequent time-dependent decrease in cell proliferation up to 21 days (79.2 ± 14.8%) was observed. Even though 7F2 proliferation on GGMA4/BSF-Eumel and GGMA4/HAp30 was lower than that of GGMA4, their overall cytocompatibility was confirmed (cell viability > 70%) and corroborated by fluorescence microscopy imaging (Figure 6c). After 24 h, osteoblasts exhibited a good proliferation on the bioactivated GGMA scaffolds, without exhibiting significant contact inhibition, with the best behavior achieved by GGMA4/BSF-Eumel (Figure 6c). However, above all, their inherent physiologically triggered activity, as bioactive stimuli capable of supporting cell differentiation, was assessed to explain the reduction observed in the proliferation of osteoblast cells. Alkaline phosphatase is an enzyme involved in the mineralization of bone and an early marker of osteoblast differentiation [56]. ALP activity was determined from the cell lysates of 7F2 cells grown onto GGMA4-based scaffolds after 7, 14 and 21 days. As shown in Figure 6b, no significant differences in cell differentiation were observed in cells seeded on GGMA4-based scaffolds at different time points. Conversely, BSF-Eumel and HAp gave interesting hints about their cell differentiation-triggering capability. GGMA4/HAp30 exhibited the highest ALP levels at 14 days (^#^ *p* < 0.001) compared to the other groups, confirming its osteoinduction ability. As shown by Frohbergh et al., 7F2 cells exhibited a negligible ALP activity when grown on a 2D plate, remaining stable over 21 days, and it was consistently lower when compared to cells grown on 3D crosslinked scaffolds, due to a higher surface area [57]. Since the 7F2 osteoblast cell line represents a late osteoblast phenotype, exhibiting an inherently significant mineralization with respect to other bone-like cell lines, in the presence of an external source of mineralizing bone elements, the cells guide their physiological response toward the differentiation, instead of the proliferation. Meanwhile, GGMA/BSF-Eumel sustained the osteoblast differentiation without any remarkable differences from GGMA/HAp (Figure 6b).

## 4. Conclusions

Despite the research’s limitations, the following conclusions were drawn.

The suggested method, which involved modifying GG and functionalizing GGMA, enabled the development of appropriate composite ink formulations. BSF-Eumel and HAp were considered as alternative bioactive signals to enhance the inherent features of the GGMA matrix. In particular, 3D scaffolds consisting of GGMA matrix functionalized with BSF-Eumel sub-micrometer particles were successfully obtained. The crosslinking times and BSF-Eumel concentration were optimized in order to minimize the UV absorption effect of the melanin. GGMA scaffolds reinforced with HAp were successfully printed even when using a higher nanoparticle concentration, overcoming the clustering effect.

GGMA-based scaffolds exhibited a well-organized and reproducible structure, with uniform pore distribution, and without collapsing in the z-direction. The scaffolds with 4% (*w*/*v*) GGMA and printed using 45% infill resulted in storage moduli in line with other comparable 3D-printed scaffolds. Notably, while the addition of HAp at 30% (w_HAp_/w_GGMA_) increased the storage modulus 3.5-fold more than GGMA, reaching a value of 32.5 kPa, BSF-Eumel did not affect the mechanical behavior. In addition, the selected processing conditions ensured the manufacturing of 3D scaffolds that were able to maintain their structural integrity throughout the course of 28 days with minimal alterations to their stability and swelling ratio. In particular, the GGMA4/BSF-Eumel scaffolds were more stable than the other structures, thanks to the presence of negatively charged groups along the eumelanin backbone. Finally, the proposed scaffolds proved their capability of supporting 7F2 osteoblast growth and differentiation processes. While GGMA4/BSF-Eumel induced a higher cell proliferation, reaching a maximum at 14 days, GGMA4/HAp30 led to a higher expression of ALP activity.

These findings provide interesting hints towards the use of these 3D-printed scaffolds for bone tissue engineering applications.

## Data Availability

The authors declare that all data supporting the findings of this study are available within the paper and its supplementary information; source data for the figures in this study are available from the authors upon request.

## Supplementary Materials

The following supporting information can be downloaded at: https://www.mdpi.com/article/10.3390/nano13040772/s1, Figure S1: ATR-FTIR spectra of: (a) GGMA4, BSF-Eumel and GGMA4/BSF-Eumel, (b) GGMA4, HAp30 and GGMA4/HAp30.
