# Supplementary material for: Bioactive Composite Methacrylated Gellan Gum for 3D-Printed Bone Tissue-Engineered Scaffolds
Source: Nanomaterials (Basel). 2023 Feb 19;13(4):772. doi: 10.3390/nano13040772 (PMC9963483; doi:10.3390/nano13040772)
Supplement: Supplementary file 1 [file nanomaterials-13-00772-s001.zip › nanomaterials-2197237-supplementary.pdf]

Supporting Information

Bioactive Composite Methacrylated Gellan Gum for 3D-Printed Bone Tissue-Engineered Scaffolds

Ugo D'Amora [1,*,†], Alfredo Ronca [1,*,†], Stefania Scialla [1], Alessandra Soriente [1], Paola Manini [2,3], Jun Wei Phua [4], Christoph Ottenheim [4], Alessandro Pezzella [1,3,5], Giovanna Calabrese [6], Maria Grazia Raucci [1,3] and Luigi Ambrosio [1]

[1] Institute of Polymers, Composites and Biomaterials, National Research Council, Naples 80125, Italy
[2] Department of Chemical Sciences, University of Naples Federico II, Naples 80126, Italy
[3] Bioelectronics Task Force, University of Naples "Federico II", Naples 80126, Italy
[4] Insectta, 60 Jalan Penjara, Singapore 149375, Singapore
[5] Department of Physics "E. Pancini", University of Naples Federico II, Naples 80126, Italy
[6] Department of Chemical, Biological, Pharmaceutical and Environmental Sciences, University of Messina, Messina 98168, Italy

* Correspondence: ugo.damora@cnr.it (U.D.); alfredo.ronca@cnr.it (A.R.)

†These authors equally contributed to this work

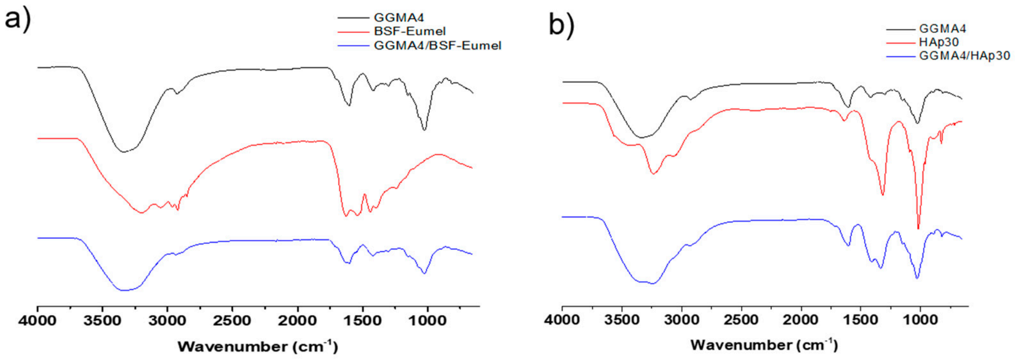

Figure S1. ATR-FTIR spectra of: a) GGMA4, BSF-Eumel and GGMA4/BSF-Eumel, b) GGMA4, HAp30 and GGMA4/HAp30.
